# Supplementary figures and images for: Optimised generation of iPSC-derived macrophages and dendritic cells that are functionally and transcriptionally similar to their primary counterparts
Source: PLoS One. 2020 Dec 17;15(12):e0243807. doi: 10.1371/journal.pone.0243807 (PMC7746299; doi:10.1371/journal.pone.0243807)

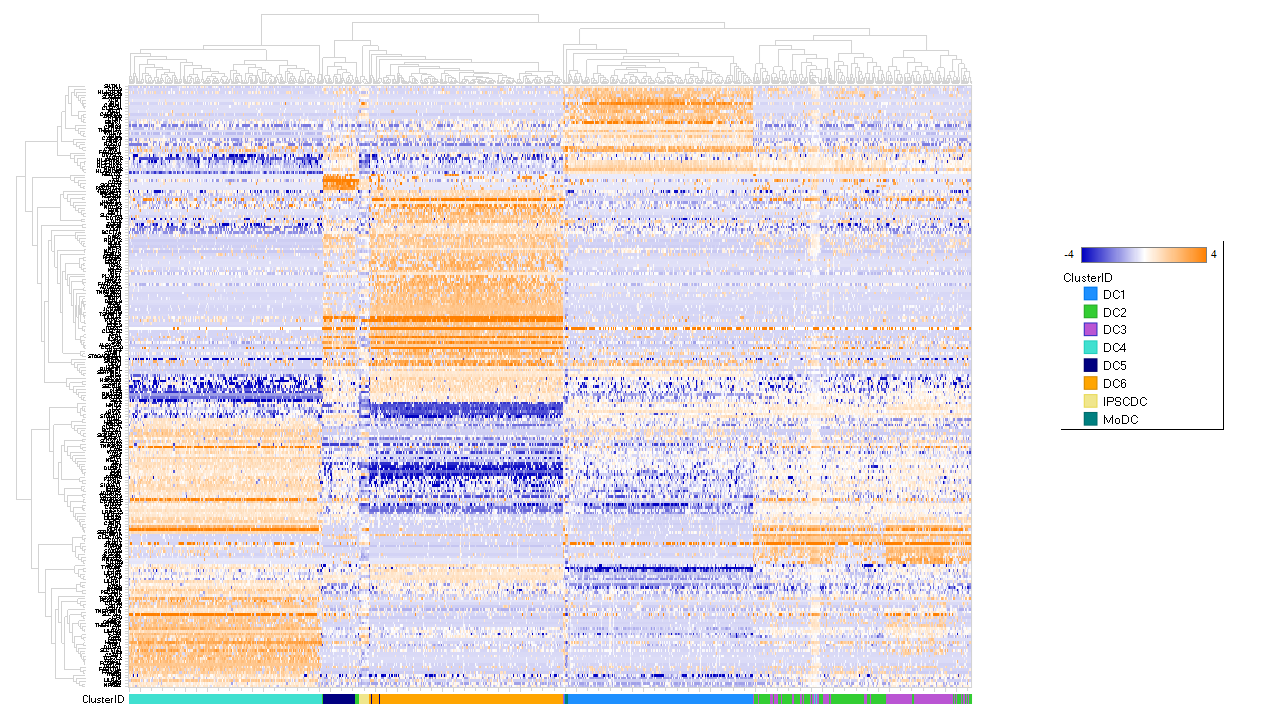

Supplement: S1 Fig — Genes are those from Villani study that discriminated the DC subgroups (S2 Table, AUC>0.85). Colours along X-axis define the sample groups (see key) and illustrate that the iPSdDC (cream) cluster between DC5 (dark blue) and DC6 (orange) while the MoDC (teal) cluster in DC1 (royal blue). (TIF) [file pone.0243807.s002.tif]

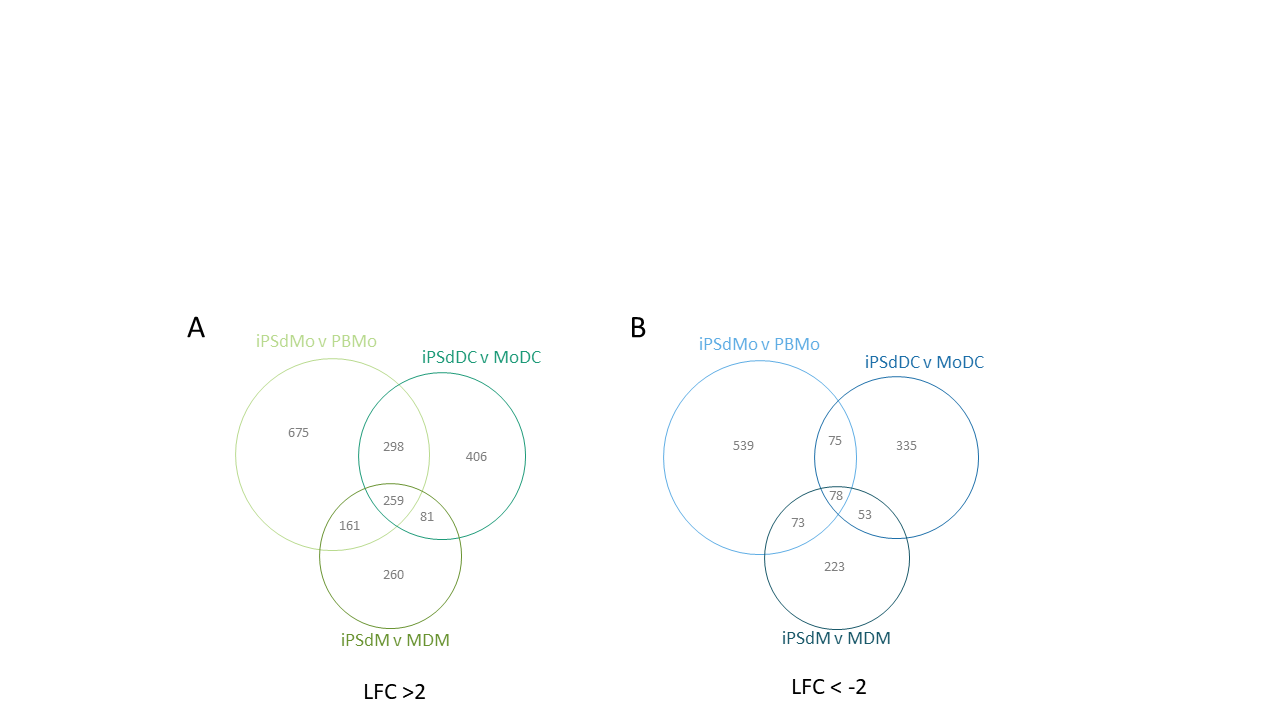

Supplement: S2 Fig — A) Up regulated genes. Gene from each comparison that passed the cut off LFC>2, padj < 0.01. B) Down regulated genes. Gene from each comparison that passed the cut off LFC< -2, padj < 0.01. (TIF) [file pone.0243807.s003.tif]
